# General and feature-based semantic representations in the semantic network

**DOI:** 10.1038/s41598-020-65906-0

**Published:** 2020-06-02

**Authors:** Antonietta Gabriella Liuzzi, Aidas Aglinskas, Scott Laurence Fairhall

**Affiliations:** 1 Center for Mind/Brain Sciences, University of Trento, Trento, 38068 Italy; 2 Department of Psychology, Boston College, Boston, 02467 USA

**Keywords:** Neuroscience, Cognitive neuroscience, Language

## Abstract

How semantic representations are manifest over the brain remains a topic of active debate. A semantic representation may be determined by specific semantic features (e.g. sensorimotor information), or may abstract away from specific features and represent generalized semantic characteristics (general semantic representation). Here we tested whether nodes of the semantic system code for a general semantic representation *and/or* possess representational spaces linked to particular semantic features. In an fMRI study, eighteen participants performed a typicality judgment task with written words ﻿drawn from sixteen different categories. Multivariate pattern analysis (MVPA) and representational similarity analysis (RSA) were adopted to investigate the sensitivity of the brain regions to semantic content and the type of semantic representation coded (general or feature-based). We replicated previous findings of sensitivity to general semantic similarity in posterior middle/inferior temporal gyrus (pMTG/ITG) and precuneus (PC) and additionally observed general semantic representations in ventromedial prefrontal cortex (PFC). Finally, two brain regions of the semantic network were sensitive to semantic features: the left pMTG/ITG was sensitive to haptic perception and the left ventral temporal cortex (VTC) to size. This finding supports the involvement of both general semantic representation and feature-based representations in the brain’s semantic system.

## Introduction

Conceptual and semantic knowledge are fundamental aspects of human cognition and the investigation of the neural substrates underlying these processes is an ongoing topic of research in the cognitive neurosciences. Although current evidence has demonstrated that semantic knowledge is represented in a distributed manner over the brain^1,2^, the manner in which semantic representation is manifest remains a topic of active debate.

The association damage to the anterior temporal lobe primary progressive aphasia, herpes encephalitis and lesions led to an emphasis of this brain regions as a critical locus for semantic processing^3,4^. However, functional neuroimaging has suggested a broader range of regions are involved in semantic processing^5^. A meta-analysis of 120 studies^6^ identified a “general semantic network” - a left-lateralized network consisting of seven brain regions that were activated in a variety of semantic tasks: angular gyrus, lateral and ventral temporal cortex, ventromedial prefrontal cortex, inferior frontal gyrus, dorsal medial prefrontal cortex and the posterior cingulate gyrus. However, not all brain regions activated in semantic tasks necessarily represent semantic content, for instance regions may control access to semantic information rather than contain that information themselves^2,7,8^. Starting from this assumption, Fairhall and Caramazza (2013)^9^ identified a set of regions representing semantic content by means of Multivariate Pattern Analysis (MVPA) and Representational Similarity Analysis (RSA)^10^. The authors showed that a left-lateralized network – consistent with the general semantic network - was not only recruited in a typicality judgmental task, but was also able to distinguish among the conceptual representation of object categories, generalizing across input-modality (written words or pictures). In addition, among all regions composing such network, the ﻿posterior middle/inferior temporal gyrus (pMTG/ITG) and the posterior cingulate/precuneus (PC) were the only two regions showing a semantic similarity effect: similarity between neural activity pattern of pMTG/ITG and PC were representative of the semantic distance between object categories. Accordingly, Fairhall and Caramazza (2013)^9^ suggested that PC and pMTG/ITG are candidate regions for the supramodal representation of the conceptual properties of objects. However, representations in several other regions were shown to be sensitive to object category, but did not conform to a general semantic representational space. It is possible that representations in these regions are related to particular semantic features, or sets of semantic features.

Object categories are frequently characterized by specific sensory and motor information (semantic features). The embodied theories of semantics claim that the semantic processing of a concept determines the re-activation of sensory and motor information produced when the referent of a word or a sentence is actually experienced^11–13^. In addition to sensory and motor information, a more recent model - the Grounded Representation in Action, Perception and Emotion Systems (GRAPES) model^14^ - suggested that the processing of a concept determines the re-activation of emotional information as well. In line with this view, it has been proposed that sensory-modality representations are located near corresponding sensory, motor and emotion networks, and by receiving bottom-up input in their modality and top-down input from other modalities, code lower-level representations. These modality-specific representations converge in higher-level cortices which bind representations from two or more modalities and code supramodal representations^15,16^.

Fernandino *et al*.^16^ investigated the role played by ﻿higher level areas by examining the activation associated with five sensorimotor features, during explicit retrieval of such sensorimotor features of written words. The results suggested a hierarchical organization of semantic memory where bimodal, trimodal and polymodal regions bind two, three or several sensorimotor information respectively. Polymodal regions, that is regions activated by all 5 sensorimotor information^16^, corresponded to high-level cortical hubs, such as ﻿angular gyrus, precuneus/posterior cingulate/retrosplenial cortex, parahippocampal gyrus, and medial prefrontal cortex.

Thus, on one hand it seems that semantic content may be intrinsically determined by sensorimotor information of the concept (feature-based representation) and on the other that some semantic representations are abstracted away from specific features and represent generalized semantic characteristics (general semantic representation). The current experiment aims at *(1)* replicating Fairhall and Caramazza (2013)’s findings and *(2)* probing whether different semantic features drive representation across nodes of the semantic system. We want to determine whether brain regions sensitive to semantic content, and that are able to distinguish between semantic categories, *(a)* code for a general semantic representation only, *(b)* code for a general semantic representation *and* possess representational spaces linked to particular semantic features, *(c)* code for particular semantic features. While the sensitivity to semantic content will be addressed by means of MVPA, the type of semantic representation (general *and/or* feature-based) will be addressed by means of RSA: By extracting information from multi-dimensional scale and by separating data into different categories, MVPA allows to determine the brain response patterns that contain semantic content. By quantifying the strength of the similarities among different neural patterns, RSA allows to identify brain regions whose brain activity is sensitive to the conceptual similarity of categories encoded (semantic representations)^10^. Secondly, we will investigate whether regions outside the semantic network are sensitive to semantic features by adopting a searchlight-RSA approach.

## Materials and Methods

### Participants

Twenty right-handed, native Italian participants (mean age M = 25.8 SD = 4.47, 11 females) took part in the study. Participants had normal, or corrected to normal, vision and were free from neurological disorders. All procedures were approved by the University of Trento Human Research Ethics Committee on the Use of Human Subjects in Research and the experiments were performed in accordance with the approved guidelines. Participants confirmed that they understood the experimental procedure and gave their written informed consent. Two subjects were removed from the analysis due to technical problems during fMRI acquisition.

### Stimulus set

An objective, data-driven strategy was employed to generate the object categories used in this study. Original stimulus selection was corpus-based. From a large text corpus (Wikipedia), we computed the co-occurrence frequency of 1481 concrete nouns (called “targets”) with 6773 words (called “features) consisting of common verbs, adjectives and nouns others than the 1481 targets. Word occurrences were log transformed, and the correlation distance (1-r) between targets was taken as a measure of word relatedness. Hierarchical cluster analysis grouped nouns into cohesive semantic categories, among which 17 were chosen. Each semantic category consisted of 24 items, which were translated to Italian. The semantic category “Leisure” containing nouns pertaining to items used for entertainment (‘radio’, ‘movie, ‘song’, ‘journal’, etc.) was removed due to low subjective consistency between the items. The final stimulus set consisted of 16 semantic categories (Clothes, Music Instruments, Tools, Households, Materials, Transports&Movement, Animals-Water, Animals-Insects, Animals-Domestic, Animals-Wild, Animals-Birds, Fruits&Vegetables, Foods&Drinks, Flora, Outdoors and Bodyparts).

### Semantic model

**Figure 1:**
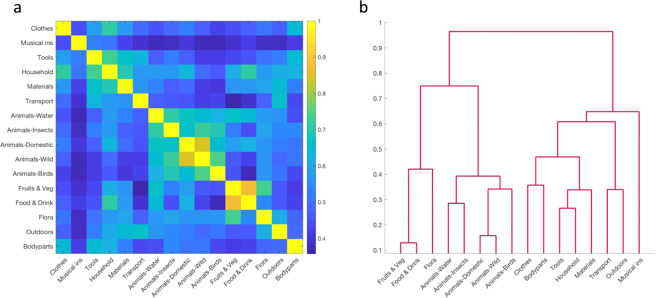
Semantic model. (**a**) Semantic model representing the semantic similarity between 16 categories. (**b**) Dendrogram of the 16 semantic categories.

The semantic model was created using word embeddings generated via the word2vec word representation models trained on the Italian Wikipedia^17^. Briefly, for each stimulus an embedding vector containing 300 values was extracted and the correlation between embeddings was used to calculate item similarity. Item similarity values were then averaged across items within a category to create the category similarity model (Fig. 1).

### Task and experiment details

**Figure 2:**
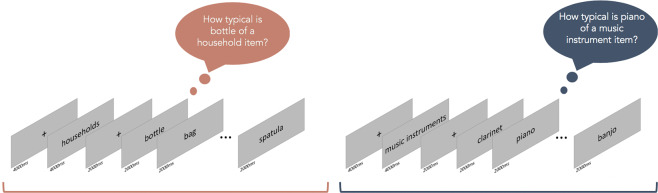
Task. Typicality judgmental task: Schematic presentation of 2 blocks related to households and music instruments category. Each block was repeated three times across runs.

18 subjects performed a blocked fMRI experiment. Each block started with a fixation cross (duration 4 s), followed by the name of the category (e.g. “Household items”) (duration 4 s). Next, an additional fixation cross appeared on the screen for 2 s, followed by a written word representing a concept belonging to the category (e.g. “Spatula”). During stimulus presentation (duration 2 s), participants were asked to read a word and rate how typical it was for the given category (How typical is spatula of a household item). Participants rated the items on a scale from 1 (low typicality) to 4 (high typicality) using a button box (Fig. 2).

The entire experiment consisted of 54 blocks divided in 2 sessions: 17 category blocks, one for each semantic category, and one block for a control condition which consisted of a 1-back matching task with function words. Each block was composed of eight stimulus presentations. Each block was presented three times, so that the 24 concepts belonging to the same category were presented once for each subject.

### Features and feature-based models

In order to measure the extent to which semantic categories rely on the relevance of specific features, eighteen new subjects performed a behavioral task. The task started with a feature-specific question aimed at focusing the participants’ attention on the relevance of each feature for that specific concept (e.g. How easily the following items can be recognized by touch?). The question was followed by a list of 64 concepts: 4 concepts for each semantic category so that, across the 18 subjects tested, each concept was rated 3 times. Subjects were asked to place the concepts on a line from low to high values/relevance. Subjects were explicitly instructed not to order the concepts from 1 to 64, but to place the category on the arrow according to the main criteria that the distance between categories was representative of the similarity (or dissimilarity) between categories for feature’s relevance. Each subject performed the task twelve times, once for each feature. Because of 5 out of 12 feature questions were interpreted inconsistently across participants, seven features were selected for the final feature dataset: Manipulation, haptic perception, color, taste, size, animacy and sound.

*Representational dissimilarities matrices for each feature were* based on the Euclidean distance between semantic categories.

### Image acquisition

A SIEMENS Prisma scanner (field strength - 3 Tesla) with a 64-channel head coil provided structural and functional images. Total of 720, T2* weighted EPI scans (voxel size - 3 mm, isotropic) were acquired during the experiment. The experiment was split into 2 runs, each lasting 12 minutes (360 volumes). Functional scans were AC/PC aligned, and 30 axial slices (gap - 0.6 mm, TR = 2000ms, TE = 28 ms, image matrix = 64 64, flip angle = 75, 108 mm brain coverage) were collected for each volume. For each subject, a high resolution T1 weighted MPRAGE (1 mm, isotropic) anatomical scan (208 sagittal slices, flip angle 12, TR - 2140 ms, TE - 2.9 ms) was also acquired. Stimuli were presented on a 42”, MR-compatible Nordic NeuroLab LCD monitor positioned at the back of the magnet bore that participants saw through a mirror in front of them. Stimuli were presented using a custom PsychToolBox 3 script running on top of Matlab R2017b.

## Data Analysis

### Preprocessing

Data were pre-processed with Statistical Parametric Mapping - SPM12 ﻿(Wellcome Trust Centre for Neuroimaging, University College London, UK). Functional images were realigned and resliced and a mean functional image was created. Next, the structural image was co-register with the mean function image and segmented. Functional images were normalized to the Montreal Neurological Institute (MNI) T1 space, resampled to a voxels size of 2 × 2 × 2 mm^3^ and spatially smoothed with 8 mm FWHM kernel. Subject specific response estimates (beta weights) were derived by fitting a general linear model (GLM) to the data with 54 blocks. Estimated motion parameters from the realignment procedure were included in the model as regressors of no interest.

### Whole-brain MVPA

Brain regions sensitive to semantic content were identified by means of a whole-brain MVPA. Subject-specific β weights were used as input of a whole-brain Multivoxel Pattern Analysis (MVPA). ﻿For each voxel in the brain, a spherical neighborhood of β values was defined with a variable radius including the 200 voxels nearest to the center voxel. A searchlight analysis was performed by using a linear discriminant analysis (LDA) classifier as implemented in CoSMo MVPA (http://www.cosmomvpa.org/)^18^. In order to compute classification accuracies, for each subject a classifier was trained on 32 β maps (two for each semantic category) and tested on 16 β maps (one for each semantic category) by means of a cross-validation measure with LDA classifier. A total of 3 iterations for each subject were performed: one for each repetition of the semantic categories. For each searchlight, accuracy scores from different iterations were averaged and the classification accuracy value was summarized at the center voxel of the sphere. To attenuate spatially non-correlated noise while preserving spatially correlated signal, the resulting subject-specific accuracy maps were smoothed with 6 mm FWHM kernel^9,19–21^ and entered into a one-sample *t* test. In order to exclude voxels with relatively low signal-to-noise ratio, a group-level mask (see section “Parametric analysis”) was used as explicit mask.

### VOI definition

VOIs were extracted from the whole-brain MVPA. Only significant clusters at P_FWE-CORR_ (cluster level) <0.001 were selected. For each local maxima a sphere with variable radius of 20 mm was created. In order to exclude voxels where no significant decoding accuracy was detected, each VOI was intersected with the whole-brain MVPA map.

### Representational similarity analysis

In order to determine whether regions sensitive to object category contained a representational space consistent with a general semantic representation, a classical representational similarity analysis (RSA) was applied. For each subject and for each VOI, a fMRI Representational Dissimilarity Matrix (RDM) was computed by extracting the 16 β weights patterns related to the 16 semantic categories (matrix 16xN.voxels) and computing a pair-wise correlation (similarity matrix 16 × 16). As each category was presented three times during the entire experiment, 3 fMRI dissimilarity matrices - one for each repetition/observation - were computed. For each subject, the 3 dissimilarity matrices were averaged and the resulting matrix was correlated with the semantic model. The significance of the results was performed by means of One-Sided *t*-Test.

Secondly, RSA was applied for addressing whether the same brain regions contained representational spaces linked to particular semantic features. For each feature, the fMRI RDM was correlated with a feature dissimilarity model partialling out all other features.

### Searchlight RSA

Sensitivity of brain regions to specific semantic features, within and outside the semantic network, was addressed by means of a searchlight RSA approach. For each subject and for each voxel of the brain, a sphere of 200 voxels was created. 16 β weights patterns related to the 16 semantic categories were extracted and a correlation between each pair of category vectors was computed, as implemented in CoSMo MVPA (http://www.cosmomvpa.org/)^18^. The obtained fMRI RDM was correlated with the model RDM. The correlation value was summarized at the center voxel of the sphere. The resulting three correlation maps, one for each repetition of the semantic category, were then averaged and smoothed with 6 mm FWHM kernel. Finally, smoothed data were entered into a one-sample *t* test with a group-level mask (see section “Parametric analysis”) as explicit mask. Significance was set at voxel-level inference threshold of uncorrected *p* < 0.001 combined with cluster-level inference of *p* < 0.05 corrected for the whole brain volume.

### Parametric analysis

In order to investigate whether sensitivity to semantic features is reflected in regional response magnitude - rather than subtle differences between voxels - a univariate analysis was performed. For each category, feature mean ratings were obtained by averaging items’ ratings. 7 t-contrasts were entered in a univariate second-level analysis, one for each feature: for each category, the regressors were weighted with feature mean ratings converted in normalized z-scores. Separate parametric analyses for each feature determined whether the bold response produced by each category varied parametrically with the importance of this feature for each category.

## Results

### Behavioral results

Across all categories, participants took on average M = 1101 ms, SD = 162 ms to respond, and the average typicality rating was M = 2.98, SD = 0.54, indicating moderate-to-high typicality of most items. Across 16 experimental categories, there were significant differences in reaction times, F(15,225) = 3.952, p < 0.001. Participants reacted the fastest when rating outdoor scenes (M = 1054 ms, SD = 125 ms) and slowest when rating tools (M = 1191 ms, SD = 136 ms). On average, however, difference in reaction times between living (n = 8) / non-living (n = 8) category domains, t(15) = 0.94,p = 0.362. Typicality ratings differed across 16 categories, F(15,225) = 15.659, p < 0.001. Items in domestic-animals category were rated as being less typical than items in other categories (Table 1). Furthermore, to see whether RSA analyses could be confounded by reaction times, we compared reaction time similarity matrix, and category semantic similarity - there were no significant correlation (r = −0.01, *p* = 0.87).

**Table 1:** Behavioral data - Reaction times and Typicality ratings. Average reaction times and deviations in milliseconds (ms). Average typicality scores, which ranged from 1 to indicate low typicality, to 4 indicating high typicality.

Category	Reaction time (ms)		Typicality rating (low (1) – high (4))		
	Mean	SD	Mean	SD	
Clothes	1082	199	2.98		0.39
Musical Instruments	1052	174	3.04		0.49
Tools	1191	136	2.77		0.49
Households	1105	162	3.12		0.44
Materials	1117	162	2.96		0.41
Transports&Movement	1087	154	3.05		0.46
Animals-Water	1090	133	2.99		0.60
Animals-Insects	1143	168	3.14		0.38
Animals-Domestic	1124	124	2.13		0.35
Animals-Wild	1186	159	2.68		0.44
Animals-Birds	1084	148	2.91		0.56
Fruits & Vegetables	1091	150	3.03		0.54
Foods & Drinks	1039	138	3.21		0.47
Flora	1113	147	3.18		0.43
Outdoors	1054	125	3.28		0.33
Bodyparts	1059	192	3.24		0.61

### Whole-brain MVPA and VOI definition

**Figure 3:**
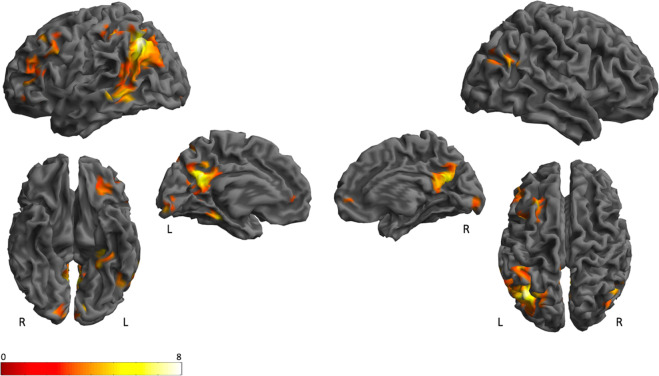
Whole-brain MVPA. 3D rendering, ﻿sagittal and axial slices, showing significant accuracy for a whole-brain MVPA at voxel-level inference threshold of uncorrected p < 0.001 combined with cluster-level inference of p < 0.05 corrected for the whole brain volume, extent threshold >202 voxels. Colors represent low (red) and high (yellow) t-values.

A whole-brain MVPA revealed ten clusters sensitive to semantic category: a cluster that extended from the left intra parietal sulcus (IPS) to the posterior middle/inferior temporal lobe (pMTG/ITG) through the angular gyrus (AG); the precuneus (PC); the right AG; the left dorsolateral prefrontal cortex (PFC); the left ventral-temporal cortex (VTC); the left inferior frontal gyrus (IFG); the left and right V1; the left lateral orbital frontal cortex (OFC); the left ventromedial PFC and the left lateral PFC (Fig. 3; Table 2).

**Table 2:** MVPA: Clusters showing significant accuracy for a whole-brain MVPA at voxel-level inference threshold of uncorrected *p* < 0.001 combined with cluster-level inference of *p* < 0.05 corrected for the whole brain volume.

	MNI coordinates			Extent	*P*FWE-CORR(Cluster level)
	*x*	*y*	*z*		
L posterior parietal-temporal cortex	−26	−64	46	3829	0.000
PC	−4	−56	28	2341	0.000
R AG	58	−60	24	291	0.009
L VTC	−32	−32	−16	309	0.007
L IFG	−50	40	6	519	0.000
L dorsolateral PFC	−28	24	36	367	0.003
L and R V1	18	−90	−4	462	0.001
L lateral PFC	−44	12	32	221	0.027
L ventromedial PFC	−8	44	2	202	0.038
L lateral OFC	−28	28	−12	234	0.022

Extent refers to the number of 2 × 2 × 2 mm^3^ voxels. *Abbreviations*: L: left; R: right; PC: Precuneus; AG: angular gyrus; VTC: ventral temporal cortex; IFG: inferior frontal gyrus; PFC: pre-frontal cortex; OFC: orbitofrontal cortex.

Twelve local maxima were extracted from a whole-brain MVPA resulting in 12 VOIs with a cluster size that ranged between 202 and 787 voxels (Table 3).

**Table 3:** VOIs extracted from the whole-brain MVPA.

VOI	Local Maxima			Size
	*x*	*y*	*z*	2 × 2 × 2 mm^3^
L pMTG/ITG	−58	−52	−10	555
L AG	−52	−64	28	787
L IPS	−26	−64	46	669
PC	−4	−56	28	707
R AG	58	−60	24	202
L VTC	−32	−32	−16	307
L IFG	−50	40	6	501
L dorsolateral PFC	−28	24	36	374
L and R V1	18	−90	−4	294
L lateral PFC	−44	12	32	225
L ventromedial PFC	−8	44	2	202
L lateral OFC	−28	28	−12	234

Size refers to a 2 × 2 × 2 mm^3^ voxels. Abbreviations: L: left; R: right; PC: Precuneus; AG: angular gyrus; VTC: ventral temporal cortex; IFG: inferior frontal gyrus; PFC: pre-frontal cortex; OFC: orbitofrontal cortex.

### Representational similarity analysis – neural and general semantic similarity

A priori pMTG/ITG and PC^9^, which are also part of the whole brain MVPA, revealed significant semantic similarity effects in both regions (pMTG/ITG: r = 0.07; *p* = 0.002; PC: r = 0.07; *p* = 0.008). Moreover, the effect remained significant after regressing out all sensory-motor features (pMTG/ITG: *p* = 0.03; PC: *p* = 0.007). Significant effects were also seen in ventromedial PFC (r = 0.09, p = 0.002), which survived Bonferroni correction for number of regions. Weaker effects that would not survive correction were present in lateral OFC (r = 0.07, *p* = 0.007), right AG (r = 0.07; *p* = 0.01), left AG (r = 0.05; *p* = 0.02) and left VTC (r = 0.05; *p* = 0.03) (Table 4).

**Table 4:** RSA for semantic effects, reaction times and typicality ratings.

	Semantics		Reaction Times	Typicality Ratings
	r	*p-value*	*p-value*	*p-value*
L pMTG/ITG	**0.07**	**0.002**	0.26	0.29
L ventromedial PFC	**0.09**	**0.002**	0.79	0.31
L lateral OFC	0.07	0.007	0.37	0.41
PC	0.07	0.008	0.17	0.36
R AG	0.07	0.01	0.58	0.43
L AG	0.05	0.02	0.98	0.37
L VTC	0.05	0.03	0.98	0.29
L IFG	0.03	0.16	0.59	0.04
L dorsolateral PFC	0.02	0.17	0.09	0.08
L and R V1	0.01	0.29	0.19	0.63
L lateral PFC	0.003	0.46	0.75	0.29
L IPS	−0.01	0.68	0.97	0.06

Values surviving a Bonferroni correction for number of regions at *p* < 0.05 are reported in bold. *Abbreviations:* L: left; R: right; pMTG/pITG: posterior middle and inferior temporal gyrus; PFC: pre-frontal cortex; OFC: orbitofrontal cortex; PC: precuneus; AG: angular gyrus; VTC: ventral temporal cortex; IFG: inferior frontal gyrus; IPS: inferior parietal sulcus.

In order to verify the specific semantic nature of the semantic similarity effects detected in the above-mentioned brain regions, we tested whether reaction times and typicality ratings played a role in such effects. Both behavioral RDMs were obtained by first averaging the reaction times and the typicality ratings across subjects for all categories, respectively, and then calculating the absolute point-wise-distance between categories. RSA was performed between the fMRI RDM and the RDM for reaction times and typicality ratings, respectively. The RDM for reaction times did not correlate with the fMRI RDM, in any of the regions tested. A significant correlation was detected between the RDM for typicality ratings and fMRI RDM in the left IFG (*p* = 0.04) (Table 4).

### Representational similarity analysis – neural and feature-based similarity

**Figure 4:**
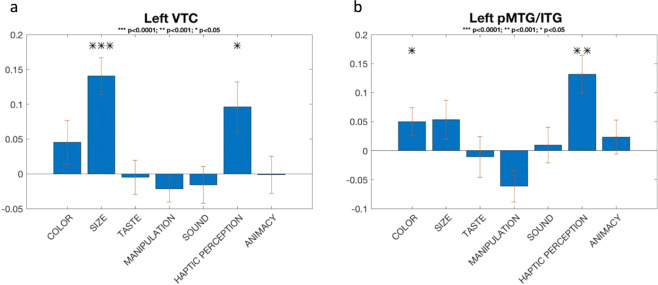
Features-based RSA. (**a**) Each bar shows an average correlation across subjects between fMRI-RDM for left VTC and feature-RDM partialling out all other features. (**b**) Each bar shows an average correlation across subjects between fMRI-RDM for left pMTG/ITG and feature-RDM partialling out all other features. Error bar: Standard Error of the Mean (SEM).

Sensitivity of the selected brain regions for features was investigated by means of RSA. For each feature tested, all remaining features were partialled out. While the left VTC was sensitive to size (r = 0.14, *p* = 0.00003) (Fig. 4a), the pMTG/ITG was sensitive to haptic perception (r = 0.13, *p* = 0.0004) (Fig. 4b). These effects remained significant after a Bonferroni correction for number of features. At more lenient uncorrected thresholds, significant effects were present for haptic perception at *p* < 0.05 in left VTC (r = 0.1, *p* = 0.008) and left IPS (r = 0.07, *p* = 0.01). Left IFG and left ventromedial PFC were both sensitive to sound (r = 0.07, *p* = 0.03; r = 0.1, *p* = 0.02, respectively) and color (r = 0.05, *p* = 0.05; r = 0.07, *p* = 0.02, respectively), while left pMTG/ITG (r = 0.05, *p* = 0.03) and the dorsolateral PFC were sensitive only to color (r = 0.06, *p* = 0.03). Finally, the left OFC was sensitive to taste (r = 0.1, *p* = 0.03) and sound (r = 0.07, *p* = 0.04).

When the semantic similarity effect in left pMTG/ITG was recomputed by partialling out the sensitivity to haptic perception, the effect was still significant (*p* = 0.05). Unlike the pMTG/ITG, the semantic similarity effect in left VTC no longer reached significance (*p* = 0.2) after partialling out the sensitivity to size.

### Searchlight RSA

**Figure 5:**
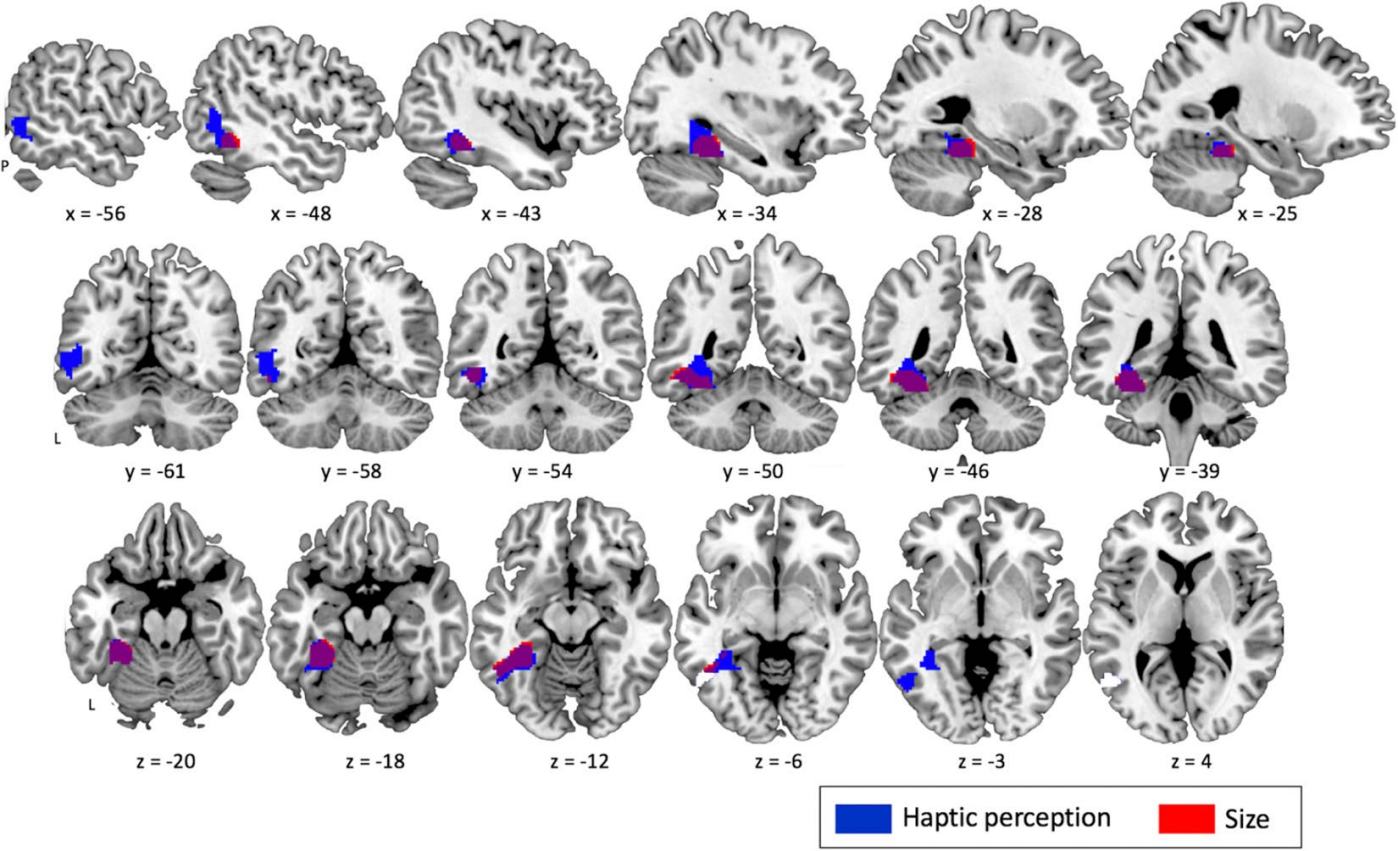
Searchlight RSA. Binary clusters sensitive to haptic perception (blue) and size (red).

To confirm the ROI analysis and determine if additional brain regions represent semantic features, we performed a whole brain RSA analysis for each feature. Among all features tested, searchlight RSA revealed 2 significant clusters that are sensitive to haptic perception and size: the left pMTG/ITG (local maxima: x = −34, y = −40, z = −20; KE = 945; PFWE-CORR = 0.001) and left VTC (local maxima: x = −30, y = −40, z = −20; KE = 565; PFWE-CORR = 0.004), respectively (Fig. 5).

### Parametric analysis

**Figure 6:**
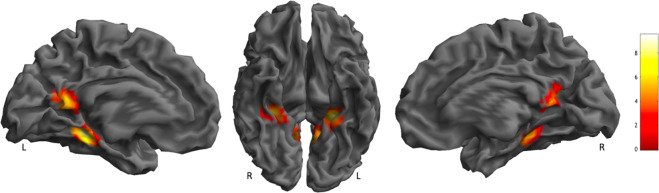
Parametric analysis. 3D rendering, ﻿sagittal and axial slices, showing significant clusters positively correlated with scores for size.

As feature-based representation was only weakly represented in the subtle voxel-wise pattern of the response, we tested whether regions weighted towards particular features were better captured in terms of the overall amplitude of their response, rather than subtle differences between voxels. A parametric analysis was conducted for each feature, whereby the univariate regressors were weighted with scores for each feature. Only the feature size, significantly predicted the amplitude of the voxel response, in left (x = −26, y = −38, z = −14; KE = 621; P_FWE-CORR_ = 0.001) and right VTC (x = 32, y = −34, z = −16; KE = 482; P_FWE-CORR_ = 0.005) and in the retrosplenial cortex (x = −10, y = −56, z = 14; KE = 1379; P_FWE-CORR_ = 0.000) (Fig. 6). The other features did not yield any significant cluster.

## Discussion

In this study, we sought to determine which regions sensitive to object category contain a representational space consistent with a general semantic representation *and/or* possess representational spaces linked to particular semantic features. MVPA identified a distributed network of generally left lateralized brain regions able to distinguish among object categories consistent with the general semantic network^6^ (Fig. 3; Table 2). RSA revealed that general semantic similarity predicted neural similarity reliably in left pMTG/ITG and PC, replicating Fairhall and Caramazza (2013), as well as in the ventromedial PFC and left OFC (Table 4). When we investigated whether different features affected the semantic representations in regions composing the semantic network, the left pMTG/ITG was sensitive to haptic perception and the left VTC to size (Fig. 4).

Convergent evidence supports the role of the pMTG/ITG as a core semantic region^6^: neuroimaging studies found significant activation in pMTG in response to tasks probing semantic processing in several input modalities (written words and pictures^22^, auditory words^23^ and tactile inputs^24^). By means of repetitive transcranial magnetic stimulation (rTMS), Hoffman *et al*.^25^ showed an involvement of the pMTG in both verbal and non-verbal semantic processing. Finally, lesion studies emphasized the relevance of this brain region in language comprehension at word level^26,27^. ﻿ However, this central role of pMTG/ITG in semantic processing does not necessitate an involvement in coding semantic representations. It might reflect retrieval/selection operations performed on semantic content^28^ or semantic control processes under the control semantic cognition (CSC) model^2^. Under this model, the pMTG, along with the left PFC, IPS, pre-SMA and ventromedial PFC, is involved in semantic control demands. This is in apparent contrast to the current finding where the conformation of neural representational spaces with semantic representational spaces strongly supports the encoding of semantic content within this region. However, many brain regions are characterized by heterogeneity of function leading to the non-mutually exclusive possibility that the pMTG have a role in both representation and control.

Similarly to pMTG/ITG, RSA results revealed a general semantic similarity effect in PC. While much evidence supports a role for the PC in semantic processing^6^, internalized “default mode”^29^, episodic memory^6,30^ and semantic control^31^, the involvement of the PC in coding conceptual representation is uncertain. However, ﻿the strong connection of the precuneus with ﻿higher association cortical and subcortical structures, the lack of direct connections between PC and primary sensory regions along with its involvement in internalized “default-mode”, led Cavanna and Trimble, (2006)^32^ to hypothesize a role of the PC in “elaborating highly integrated and associative information, rather than directly processing external stimuli”. Given the minimal episodic demands imposed by the task adopted (typicality task), the semantic similarity effect in PC seems to be in line with Cavanna and Trimble’s hypothesis: by elaborating highly integrated information, the PC does code for general semantic content. Accordingly, the strong relationship between neural and conceptual representational spaces evident in the current work, Fairhall and Caramazza (2013)^9^ as well as Liuzzi *et al*.^33^, provides compelling evidence that the PC plays also a role in conceptual representation.

Importantly, the general semantic similarity effect was tested by performing an RSA with a corpus-based semantic model. Such model provides a representation that incorporates the contribution of object features across a range of domains (both concrete and abstract) in a way that generalizes across all features. While this forms a representation less affected by any specific objects features, specific features contribute to this semantic space and may potentially effectively capture and model neural responses. We partially tested this hypothesis by regressing out all sensory-motor features used from the general semantic similarity for the pMTG and PC. As the effect remained significant in both regions, we are more confident in claiming a role of pMTG/ITG and PC in coding general semantic similarity, but with the caveat that we did not remove all possible features.

Besides pMTG/ITG and PC, a semantic similarity effect was found in the frontal cortex, namely in the ventromedial PFC and left lateral OFC. Both ventromedial PFC and lateral OFC are strongly connected to the medial temporal cortex and activated in many semantic contrasts^6,34^. With regards to the ventromedial PFC, recent evidence highlighted its contribution to reward-guided learning and decision-making^35^, semantic control demands^2^ and memory consolidation^36^. More in detail, Nieuwenhuis *et al*.^36^ proposed that the ventromedial PFC is an integrative region receiving information about value of items from ﻿perirhinal cortex and lateral orbitofrontal cortex and contextual information from ﻿parahippocampal cortex and medial prefrontal cortex. In this sense, it is a region able to ﻿represent the value of items and their associated actions based on what is known about the current context. Similarly to the ventromedial PFC, the lateral OFC plays a crucial role in emotion, decision-making^37^ as well as in semantic monitoring/discrimination tasks^34^. Although further research is needed, current results show that both ventromedial PFC and lateral OFC are strongly involved in coding general semantic representations.

Additionally, weaker semantic similarity effects were seen at lenient uncorrected threshold (*p* < 0.05) in left AG and a homologous region in the right hemisphere as well as the VTC. While caution must be taken in interpreting these effects, this seems to suggest that general semantic representation may be more widespread than previously thought^9^. The AG had been implicated in integrating conceptual information^6,38^ as well as combinatorial processing^39^. While the posterior section of the VTC observed here is frequently implicated in semantic processing^6^, no activation was detected in the anterior section of the VTC, which may result from low signal to noise in this region in the scan sequence we used^40^.

The left IFG was one of the brain regions sensitive to semantic content: it exhibited different patterns of activation for different semantic categories. No semantic similar effect was detected in this region. The left IFG has been implicated in selection^7^, semantic working memory^41^, ﻿integration between word and world knowledge^42^ and semantic control^2^. It is possible that the sensitivity to semantic category detected in this region might be due to subtle differences in semantic control demand between categories. This possibility is supported by the observation that the representational space in left IFG conformed to typicality ratings, an indirect index of control demand, rather than to semantic distance (see also^9^). A meta-analysis of 2013^43^ showed that, along with the IFG, cortical responses of pMTG and IPS correlated with semantic control demand. In the current study, no correlation was detected between the fMRI RDM for the IPS and typicality ratings RDM. With regards to the pMTG, the neural distance of the pMTG correlated with the semantic distance, but it did not significantly correlate with typicality ratings differences. As already mentioned early, current results open the possibility of a dual role of the pMTG in both representation and semantic control.

The second goal of the current experiment was the investigation of whether different semantic features drive the representation across nodes of the semantic system. Two brain regions of the semantic network were sensitive to semantic features: after partialling out all other features, the left pMTG/ITG was sensitive to haptic perception and the left VTC to size (Fig. 4).

In addition to being implicated in semantic representation^9^ and control^2^, the pMTG in also involved in hand and non-hand actions observation and imitation^44^, action perception and understanding^45^ visual action and action knowledge^46^ and tool recognition^47^ and lesions to pMTG are associated with tool related semantic deficits^5^. While our current results show a sensitivity to haptic perception, a previous study on sensorimotor features^16^ showed that the left pMTG was sensitive to sound and visual motion. Such discrepancy in sensitivity of the pMTG to specific semantic features might be explained in terms of task: while Fernandino *et al*.^16^ asked ﻿participants to decide whether the stimulus referred to something that can be experienced through the senses, we used a typicality judgment task, which does not require an explicit retrieval of sensorimotor information but encourages most prototypical semantic access for that feature.

In order to test whether the general semantic similarity effect in the left pMTG/ITG was influenced by the sensitivity of this brain region to a specific features (e.g. haptic perception), the correlation between the semantic model and the fMRI data (RSA) was recomputed by first partialling out the effect for all features and secondly by partialling out the effect for haptic perception: As the semantic similarity effect was still significant, our results prove the involvement of the left pMTG/ITG in coding general semantic relationships beyond feature representation^9^.

While the pMTG/ITG was sensitive to haptic information, the left VTC was sensitive to size. Although lateral and ventral temporal cortex are considered heteromodal regions involved in concept retrieval and supramodal integration^6^, a more recent theory - the ﻿Posterior Medial Anterior Temporal (PMAT) model^48,49^ – proposed an anatomical and functional distinction between the anterior and the posterior portion of the medial temporal cortex, each belonging to distinct cortico-hippocampal networks. According to the PMAT model, perirhinal cortex and parahippocampal cortex are core components of the anterior and the posterior portion of the medial temporal cortex, respectively. At functional level, while the anterior portion is involved in item-based memory, the posterior portion is instead implicated in spatial navigation, episodic retrieval and situational models. However, the posterior VTC is also characterized by large-object selectivity: while left PHC is sensitive to scenes^50^, to access to place-related semantic knowledge^51–53^, to salient objects that are part of specific scene^54,55^, it is also sensitive to big, compared to small, objects^56^. Interestingly, studies on blind population^57,58^ showed that such large-object selectivity of the PHC does not require visual processing or even visual experience: compared to tools and animals, large nonmanipulable objects activated the PHC in both sighted and blind people^57^. The current finding that representational spaces in this region conforms to size relationships between object-categories indicates the importance of feature-based semantic representation in this region.

While general semantic representation of multiple categories and features may be represented on a fine neural scale, allowing the simultaneous representations of multiple semantic dimensions, regions specialized for a particular feature like size may be more homogenous and their input into the representation represented in their overall response. We tested this hypothesis by means of a parametric analysis where – for each feature – the univariate regressors were weighted with normalized z-feature-scores. Besides the PHC, this parametric analysis revealed a sensitivity to size in the RSC as well. Similarly to PHC, RSC is involved in spatial navigation, scene^50,59^ as well as physical size^60^. By investigating two scene properties, namely physical size and ﻿functional clutter (the organization and quantity of objects that fill up the space), Park *et al*.^60^ demonstrated that ﻿while ﻿parahippocampal place area (PPA) was sensitive to both size and clutter, RSC showed much stronger pattern sensitivity to size only. Our parametric results were slightly more robust (PHC: peak-level T = 9.52) than a pattern-based analysis (PHC: peak-level T = 9.31), supporting the idea that parametric models seem to capture feature-based effects more than pattern-based analyses. Thus, general semantic representation may be represented in the fine scale pattern of activation within a region, while uni-dimensional features represented in a specialized region may contribute to representation through their overall level of activity.

## Conclusions

The current finding supports a model of generalized and specialized semantic representation. Firstly and importantly, we replicate previous findings of general representation of objects that conform to their overall semantic similarity in the left pMTG/ITG and PC^9^ and extend these findings to show additional representations in vmPFC. Furthermore, we report that this system is complemented by representations that are weighted towards particular features: the left pMTG/ITG is sensitive to haptic perception and the left VTC to size. The nature of feature-based and generalized semantic representation may differ: at least in the case of one feature – size - the feature is encoded within the whole region rather than subtle variation in the pattern of the response across voxels.
